# National Technology Validation and Implementation Collaborative (NTVIC): Updated guidelines for establishing Forensic Investigative Genetic Genealogy (FIGG) programs

**DOI:** 10.1016/j.fsisyn.2025.100593

**Published:** 2025-06-27

**Authors:** Ray A. Wickenheiser, Jennifer Naugle, Brian Hoey, Rylene Nowlin, Claire Glynn, Raymond Valerio, Lance Allen, Stephanie Stoiloff, Jennifer Kochanski, Mary Beth Schubert, Melissa Haas, Dixie Peters, Rachel Oefelein, Rockne Harmon, Barbara Llewellyn, Trisha Kacer, Blake Patel, Paul Panther, Colleen Fitzpatrick, Garry Bombard, Alison Sears, David Mittelman, Stacey Baker, Stephanie M. Fullerton, Jessica Koong, Meredith Chambers, Melisa Wittkowske, Ken Carrierre, Nichole Schwedelsky, Ted Liomanis, Michael Kelly, Stephen Smith, Jonathan Nauman, Tom Cober

**Affiliations:** a New York State Police Crime Laboratory System, Albany, NY, USA; b Ray Wickenheiser Forensic Consulting, Youngsville, LA, USA; c Wisconsin Department of Justice Division of Forensic Sciences, USA; d Missouri Highway Patrol Crime Laboratory Division, USA; e Idaho State Police Forensic Services, USA; f University of New Haven, Henry C Lee Institute of Forensic Science, USA; g Queens District Attorney's Office, USA; h Colorado Bureau of Investigation Forensic Services, USA; i Miami-Dade Police Department, Forensic Services Division, USA; j Arizona Department of Public Safety, USA; k Retired Sacramento District Attorney, USA; l Bode, USA; m Center for Human Identification, University of North Texas Health Science Center at Fort Worth, USA; n DNA Labs International, USA; o Forensic/Cold Case Consulting, USA; p Relationship Testing, AABB, USA; q Texas Department of Public Safety, USA; r Hillsborough County Medical Examiner Department, USA; s Idaho Attorney General's Office, USA; t Identifinders International, USA; u Loyola University Chicago and Illinois State Police, USA; v NSW Police Force, Australia; w Othram, USA; x Palm Beach Sheriffs Office, USA; y University of Washington School of Medicine, Bioethics and Humanities, USA; z QIAGEN (GEDmatch), USA; aa West Virginia State Police Forensic Laboratory, USA; ab Calgary Police Service, Canada; ac Toronto Police Service, Canada; ad York Police Service, Canada

## 1 Introduction

On February 15, 2023, the National Technology Validation and Implementation Collaborative (NTVIC) Forensic Investigative Genetic Genealogy (FIGG) Technical Validation Working Group (TVWG) Subcommittee for Public Entity Policy and Procedure published an article in the journal Forensic Science International: Synergy entitled “National Technology Validation and Implementation Collaborative (NTVIC) Policies and Procedures for Forensic Investigative Genetic Genealogy (FIGG)” [1]. This document provided guidelines and considerations for public and private crime laboratories and investigative agencies exploring the establishment of a forensic investigative genetic genealogy (FIGG) program [1]. On November 21, 2023, and update publication in the journal Forensic Science International: Synergy entitled “National Technology Validation and Implementation Collaborative (NTVIC): Guidelines for establishing Forensic Investigative Genetic Genealogy (FIGG) programs” incorporated feedback provided to improve on the previous publication [2]. Additional input and experience of additional casework, court cases, and a broader base of consultation has provided this updated third version.

While this document was prepared by members of the Policy and Procedures Subcommittee of the FIGG-TVWG and the Canadian FIGG Working Group, inclusion of an individual's name does not imply full agreement with every recommendation or statement. The content represents a consensus of perspectives and contributions from the group as a whole.

**Table:** 

Member	Organization
Ray Wickenheiser, DPS (Co-chair)	Director, New York State Police Crime Laboratory System (retired)^1,2,3^
Jennifer Naugle (Co-chair)	Deputy Administrator, Wisconsin Department of Justice Division of Forensic Sciences^1,2^
Brian Hoey	Director, Missouri Highway Patrol Crime Laboratory Division^1,2^
Rylene Nowlin	Idaho State Police Forensic Services^1,2^
Claire Glynn, Ph.D	University of New Haven, Henry C Lee Institute of Forensic Science^1,2^
Raymond Valerio	Assistant District Attorney, Queens District Attorney's Office^2^
Lance Allen	Colorado Bureau of Investigation Forensic Services^2^
Stephanie Stoiloff	Chief, Miami-Dade Police Department, Forensic Services Division^2^
Jennifer Kochanski	Arizona Department of Public Safety^2^
Anne Marie Schubert	Retired Sacramento District Attorney^2^
Melissa Haas	Bode
Dixie Peters	Center for Human Identification, University of North Texas Health Science Center at Fort Worth
Rachel Oefelein	DNA Labs International
Rockne Harmon	Forensic/Cold Case Consulting
Barbara Llewellyn, Ph.D	Program Manager, Relationship Testing, AABB
Trisha Kacer	Texas Department of Public Safety
Blake Patel	Hillsborough County Medical Examiner Department
Paul Panther	Special Deputy, Idaho Attorney General's Office
Colleen Fitzpatrick	Identifinders International
Dr. Garry Bombard	Loyola University Chicago and Illinois State Police (retired)
Alison Sears	NSW Police Force
David Mittelman, Ph.D	Othram
Stacey Baker	Palm Beach Sheriffs Office
Dr. Stephanie M. Fullerton	University of Washington School of Medicine, Bioethics and Humanities
Jessica Koong	QIAGEN (GEDmatch)
Meredith Chambers	West Virginia State Police Forensic Laboratory
Dr. Melisa Wittkowske	Wisconsin Department of Justice Division of Forensic Sciences
Ken Carrierre	Calgary Police Service^3^
Nichole Schwedelsky	Calgary Police Service^3^
Ted Liomanis	Toronto Police Service^3^
Michael Kelly	Toronto Police Service^3^
Stephen Smith	Toronto Police Service^3^
Jonathan Nauman	York Police Service^3^
Tom Cober	York Police Service^3^

^1^ Denotes an original committee member.

^2^ Denotes a committee member participating in the previous version.

^3^ Denotes a Canadian FIGG Working Group member.

## 2 Scope

Forensic Investigative Genetic Genealogy (FIGG) utilizes an evidentiary single nucleotide polymorphism (SNP) deoxyribonucleic acid (DNA) profile generated by next generation sequencing (NGS) and established genetic and genealogical research methodologies to generate investigative leads in unsolved investigations such as missing and unidentified persons investigations and violent crimes [[1], [2], [3], [4], [5], [6]]. On February 15, 2023, the NTVIC published an article entitled “National Technology Validation and Implementation Collaborative (NTVIC) Policies and Procedures for Forensic Investigative Genetic Genealogy (FIGG)” to summarize existing published guidance, including the Scientific Working Group for DNA Analysis Methods (SWGDAM) IGG Overview Document [7], the United States Department of Justice (US DOJ) Interim Policy [8], and the Maryland legislation [9]. The article also solicited feedback from the forensic community on a draft of proposed best practices and considerations for developing forensic genetic genealogy (FGG) SNP DNA profiles and conducting investigative genetic genealogy (IGG) in public laboratories and investigative agencies. Since the publication of the aforementioned paper [1], the state of Utah has passed the “Sherry Black Bill” [10]. In addition, multiple members of the forensic science, law enforcement, legal, and genealogical communities have provided extensive feedback on the draft guidelines. On November 21, 2023, an updated publication in the journal Forensic Science International: Synergy entitled “National Technology Validation and Implementation Collaborative (NTVIC): Guidelines for establishing Forensic Investigative Genetic Genealogy (FIGG) programs” incorporated feedback provided to improve on the previous publication [2].

Individual jurisdictions are responsible for establishing their own FIGG policies and procedures, overseeing its implementation and developing frameworks for evaluating program performance. The purpose of this paper is to provide a publicly available updated version that considers feedback from stakeholders and other interested parties regarding (1) compatibility with existing law, and (2) guidance for public and private-sector laboratories, investigative agencies and legal professions that may be developing state/provincial or local policies and procedures related to the establishment of a FIGG program. This third version is to update and augment previous NTVIC FIGG guidance with a broader base of input and in particular add additional information on privacy, security and discovery. The term FIGG Responsible Authority (FIGG RA) is used herein to refer to the body responsible for the use and oversight of FIGG in a particular jurisdiction [1,2].

The scope of this document includes guidance on:

• Terminology and definitions used during the FIGG process
• Organizational approaches for establishing a FIGG Program
• Considerations for development of FIGG policy and procedures
  • case qualification and triaging
  • utilization of genetic genealogy databases
  • obtaining third-party DNA samples
  • obtaining putative perpetrator DNA reference samples
  • uses of certain genetic information and restrictions
  • privacy/protection of information
  • retention, release and expungement of information
  • documentation
  • discovery
• FIGG Education & training
• Quality Assurance and Quality Control (QA/QC)
• FIGG Case Reporting
• FIGG Processes
• FIGG Privacy and security considerations
• FIGG Discovery
• FIGG Oversight

## 3 Guidelines

1.0 Terminology and Definitions
  1.1 “Combined DNA Index System” or “CODIS”: the program operated by the Federal Bureau of Investigation to support criminal justice DNA databases and the software used to run the databases” [11].
  1.2 “Criminal proceeding”: the adversary judicial process prosecuted by a public officer and initiated by a formal complaint, information, or indictment charging a person with an offense denominated as criminal by applicable law and punishable by death, imprisonment, or a jail sentence” [12].
  1.3 “Direct-to-consumer (DTC) genetic genealogy services”: genetic genealogy services including databases that are offered by private companies directly to members of the public and investigative agencies rather than through clinical health care providers, typically via customer access to online websites” [9].
  1.4 “DNA”: deoxyribonucleic acid.
  1.5 “DNA specimen” or “specimen”: a biological sample collected from an individual or a crime scene or collected as part of an investigation [11].
  1.6 “DNA Technical Leader”: is an individual responsible for the oversight of the quality system in an accredited forensic DNA laboratory.
  1.7 “Single nucleotide polymorphisms” or “SNP”: DNA sequence variations that occur when a single nucleotide (A, T, G or C) in a genomic sequence varies [9]. SNPs include variations that may be used to distinguish people for purposes of biological relationship testing or direct comparison purposes [1].
  1.8 “Short tandem repeat” or “STR”: a genetic location in which the polymorphism or genetic variation is based primarily on the differences in the repeat elements among individuals. STRs are the genetic markers that are used primarily in local, state, and national DNA database systems [1].
  1.9 “Familial DNA Searching” or “FDS”: a technique that deliberately searches for close biological relatives of a forensic STR DNA profile donor within an investigative database [13]. FDS typically is capable of determining close family relationships including immediate family members, i.e., parent-child, siblings.
  1.10 “Forensic Investigative Genetic Genealogy” or “FIGG”: a procedure that combines genetic testing with traditional genealogical research to generate leads in investigations of unsolved violent crimes and unidentified human remains [1].
  1.11 “Forensic Genetic Genealogy” or “FGG”: the process of developing a DNA SNP profile to be specifically uploaded into a genealogical database.
  1.12 “Informed consent” as it pertains to third parties: consent based on disclosure of the purpose of third-party DNA sample collection and procedures for storing, transferring, and destroying the sample and associated data with an opportunity to ask questions and request clarification.
  1.13 “Investigative Genetic Genealogy” or “IGG”: the investigative component of FIGG, that includes the upload of a SNP profile into a genealogical database, the creation of a family tree, and the investigation of leads [1].
  1.14 “FIGG Responsible Authority” or “FIGG RA”: the body responsible for the conducting and oversight of FIGG in a particular jurisdiction [1]. The FIGG RA may be determined by the jurisdiction within which FIGG will be applied or as determined by lawful authority.
  1.15 “Forensic sample”: biological material collected from a crime scene, person, item, or location connected to the criminal event, that is reasonably believed by investigators to have been deposited by a putative perpetrator. A forensic sample also includes the biological material from unidentified human remains (UHR) [9].
  1.16 “Putative perpetrator”: one or more criminal actors reasonably believed by investigators to have committed the crime under investigation and to be the source of, or a contributor to, a forensic sample deposited during or incident to the commission of a crime [9].
  1.17 “Reference sample”: biological material collected from a known source [9].
  1.18 “Third-party”: a person who is not a suspect in the investigation, however who may provide a lead through kinship association to identify the unknown donor of a forensic sample [9].
  1.19 “Third-party DNA testing”: a DNA analysis conducted on a collected DNA sample from an individual with their consent who is not a suspect in an investigation, in an attempt to further genealogical research to identify the putative perpetrator.
  1.20 “Unidentified human remains” or “UHR”: human remains from a person whose identity has not been established.
  1.21 “Whole genome sequencing” or “WGS”: DNA analysis that determines the order of nucleotides (A, T, G or C) in an individual's entire genome.
2.0 Organizational Approaches for Establishing a FIGG Program
  2.1 Lawful Authority: Lawful authority and activity must be established to ensure compliance with privacy requirements. This can be accomplished through establishing a FIGG Responsible Authority (FIGG RA). The FIGG RA is an investigative agency or duly appointed authority responsible for the investigation of crime and its potential prosecution. The FIGG RA will comply with all applicable privacy laws, not publishing or making available publicly the identity of the individuals included in genealogical research, except the individual eventually charged with the offense, in accordance with applicable legal documentation and guidance regarding inclusion of the charged individual.
  2.2 Accountability: Law enforcement is responsible for DNA and DNA-derived information under their control from familial DNA and genealogical searching and must adhere to privacy best practices.
    1. The FIGG RA is responsible for DNA and DNA-derived information for all familial DNA and genealogical searching under their control. This includes situations when samples are outsourced to vendors.
    2. The FIGG RA shall adhere to best practices, continuously improving its policy and methodology.
    3. The FIGG RA should review vendor practices, establish and document requirements through a Memorandum of Understanding (MOU) with all entities having access to DNA and DNA-derived information. (See 3.10 Third-party procurement).
  2.3 Each jurisdiction should designate a FIGG RA to develop a framework for organizing, overseeing, and conducting a FIGG program based on their needs and legal requirements. Some models for consideration include:
    A. Utilizing existing established processes and associated policies and procedures, namely, the laboratory (public or private) that maintains responsibility for DNA typing and/or database searching. Once an association or lead is made; law enforcement supports further investigation.
    B. Designating a coordinating group consisting of all or a subset of representatives from law enforcement, the prosecuting attorney's office, and an authorized submitting investigative agency to facilitate communication and operations of FIGG.
    C. Choosing a hybrid approach of 2.3.A and 2.3.B.
3.0 Considerations for FIGG Policy and Procedures
  Each country, state, province, region, and/or agency may develop their policies and procedures for implementation, in alignment with the individual jurisdiction's laws. Law enforcement must address the privacy of necessity and proportionality. A policies and procedures document may include, but is not limited to, the following considerations:
  3.1. Necessity dictates that:
    1. There is biological evidence from the perpetrator left at the scene of a crime which could assist in solving the crime
    2. The crime is not immediately solvable without analyzing this evidence, and
    3. The perpetrator remains at large potentially committing new crimes on the same or additional victims.
  3.2. Cases will be considered for FIGG which are major unsolved crimes against the person. The bioethical concept of proportionality requires weighing two competing interests, in the case of crime investigation, the risk to society versus individuals' right to privacy. This risk to society with the bioethical concept of proportionality dictates that FIGG may be conducted to:
    1. Fully analyze the crime scene sample with all available technology,
    2. Use the genealogical profiles from individuals with informed consent to compare to relative's profiles or to crime scene profiles, and
    3. Use publicly available information to build family trees to include a potential suspect.
  3.3. All cases that have DNA from the putative perpetrator at the crime scene or are a UHR (Unidentified Human Remains) with sufficient and suitable DNA should be considered for FIGG. Considerations include the following:
    a. Cases qualify as serious crimes against the person where public safety has been put at risk so as to require FIGG and outweigh individual's right to privacy of their publicly available genealogical information and relationships which has been aggregated in genealogical websites, databases, and other formats
    b. Samples qualify as being of sufficient quality and quantity to generate a DNA profile from the putative perpetrator
    c. Resources are in place to conduct FIGG
    d. Only genealogical data from databases permitting law enforcement searching where individuals have provided informed consent (affirmatively opted in or have the option to opt out) may be searched against the forensic profile
  3.4. Right to privacy: Perpetrator biological samples located at a crime scene are abandoned (discarded) samples, and as such the individual has no expectation of privacy for those abandoned samples. Therefore, the forensic sample can and should be fully interrogated to determine its originator to assist in solving the crime [1,2,4]. A crime scene sample is distinctly different from a sample taken from a known individual. The donor of a crime scene sample is not known; the goal is to identify them to provide investigative leads to help solve the crime. Even when matched to a known individual, samples from crime scenes should be retained in perpetuity, as they can be used to link to other crime scenes and may help to solve other unsolved crimes.
  3.5. Right to autonomy: Individuals have a right to personal autonomy over their own body, DNA, and its use. An individual can exercise their autonomy with informed consent through a process known as opting in to permit their DNA to be placed in a database to be compared to forensic sample profiles to assist law enforcement agencies to provide investigative leads to help solve crimes.
  3.6 Qualifying cases
    Each jurisdiction should develop its criteria for case qualification, including exigent circumstances that may be evaluated on a case-by-case basis. The US DOJ Interim Policy and the Maryland and Utah laws governing FIGG include some case qualification considerations.
    A. A violent crime as defined in the relevant jurisdiction [7]; or
    B. Other violent crimes and/or defined by law and/or jurisdiction; or
    C. Identification of a missing or unidentified human remains [9]; or
    D. Post-conviction testing for crimes as defined in 3.6.A, B, and C above.
  3.7 Case status
    Current guidance suggests that reasonable investigative leads must be pursued prior to pursuing FIGG [1,8,9]. Each jurisdiction should develop its criteria for case management. Exceptions to a policy may be considered on a case-by-case basis, with FIGG being applied directly and/or concurrently with other investigative techniques such as when:
    A. Biological evidence is limited, e.g., severely degraded or low input samples
    B. A CODIS search did not yield a hit or was not possible because of the sample type (e.g., rootless hair), or
    C. There is a significant and exigent risk to public safety.
  3.8 Forensic samples
    Forensic samples should be sufficient to develop a viable SNP DNA profile [1].
    A. If a forensic sample has the potential to be consumed in analysis, consideration should be given to:
      i. Applying the most appropriate analytical technique to maximize the chances of obtaining useable data.
      ii. Delaying analysis as technology improves to enable a profile to be generated. The sensitivity of DNA analysis is rapidly developing, particularly for forensic samples. Therefore, sample preservation is critical to ensure the optimum opportunity for profile generation.
      iii. Re-examining case items for additional biological samples. Original forensic analysis traditionally did not aim to harvest all biological materials from evidence items, but rather to sample materials that may provide a result. Therefore, other items may possess additional biological materials that can be successfully analyzed to generate a SNP DNA profile.
    B. Whole sample consumption is considered a last resort in forensic investigations, so must be given due consideration, have appropriate documentation and authorization, and be in compliance with federal and state/provincial laws prior to proceeding with analysis [*see Arizona v. Youngblood*, 109 S.Ct. 333 (1988)]. A separate request for whole sample consumption for FIGG purposes should be reviewed and authorized by the investigative agency, after considering input from the forensic laboratory and the prosecuting agency. All approvals from stakeholders should be retained within the case documentation. Developed policies for whole sample consumption shall be consistent with applicable law. Forensic laboratories may develop policies on consumption with their local prosecuting attorney or equivalent and provide them to submitting agencies.
  3.9 Familial DNA searching (FDS):
    Different ways to generate a lead via a DNA database include direct searching in CODIS, FDS or indirect searching in CODIS, and FIGG using public genetic genealogy databases. FDS uses STR DNA profiles and CODIS to develop investigative leads through a deliberate search for biologically related individuals [13]. An estimated 20% of individuals in an investigative databank may be siblings [14]. FDS can be conducted independently from FIGG and does not require a SNP profile. Therefore, FDS should be considered (in jurisdictions permitting such searches) for unsolved cases where an STR forensic profile has been generated prior to pursuing FIGG.
    Jurisdictions should develop triaging criteria to determine which database option(s) may be pursued, based on the sample type, the quantity/quality of DNA and the resulting STR DNA profile, the case circumstances, and the jurisdictional authority to use FDS, all of which should be considered prior to, simultaneously to, or independently of FIGG.
  3.10 Third party procurement: Law enforcement remain accountable for their use of IGG when outsourcing to FIGG services to third parties.
    1. The FIGG RA is fully responsible and accountable for the outsourcing of FIGG to third parties.
    2. The FIGG RA should use accredited vendors performing all aspects of FIGG, including genealogical researchers.
    3. Genealogical research should be accredited, which includes description and documentation of required education, training, experience and qualifications of genealogical researchers, FIGG training, competency tests, ongoing proficiency tests, validation of methods, documentation of methods, and documented case files. In some cases where warranted, genealogical accreditation may be based on evaluation of experience and qualifications obtained prior to the development and acceptance of an official accreditation process.
    4. Any individual or entity accessing or retaining case data should be ISO accredited by an ISO accredited entity.
    5. It is recommended that the FIGG RA create a MOU with vendors working on a FIGG sample that includes the above information.
    6. It is recommended that jurisdictions keep a secure facility to maintain all evidence related to both FIGG and forensic documentation.
    7. Assuming that agreements have been established and documented between the FIGG RA and a vendor via a MOU, the vendor may keep a case file to include a complete record of all procedures and analysis conducted.
    8. If a vendor is to retain data, the FIGG RA should have a policy for who is responsible for the data generated by the vendor lab and vendor genealogist. If the vendor is bought, or the vendor goes out of business, procedures and responses should be in place to ensure all data is retained and sent back to the FIGG RA. Procedures should also be in place to ensure individuals who worked on a case are available for testimony, regardless of whether they change jobs, no longer work for the vendor, and that cover other potential issues that may jeopardize testimony availability.
4.0 Program Personnel and Partners
  4.1 Roles and responsibilities for those involved in a FIGG program may, include all or a subset of the following, based on the needs of individual jurisdictions:
    A. A designated laboratory official (DLO) [8];
      i. The DLO may be assigned by forensic laboratories and investigative agencies that are implementing FIGG.
      ii. The DLO's duties may include but are not limited to; assessing case eligibility, DNA sample and profile assessment, available SNP technology options, triaging mechanisms seeking authorizations for whole sample consumption, coordination of sample outsourcing to vendor laboratories (if applicable) and overseeing the upload/deletion of SNP DNA profiles from the database(s) following completion of a case.
      iii. The DLO may function as a single point of contact acting as a liaison between law enforcement, forensic laboratories, private laboratories, genealogical researchers, justice system officials, and other FIGG stakeholders.
      iv. The DLO provides information and education to key stakeholders, ensures compliance with laboratory policy and quality standards, maintains documentation of case records and recommendations, and performs other duties similar to those a CODIS administrator position currently requires [11].
    B. An administrative representative from the vendor sequencing laboratory, if used (DNA expertise) [8];
    C. An administrative representative with genealogical research expertise [1];
    D. An investigative agency representative with access to investigative databases (metadata) and crime analysis [1];
    E. A representative from the requesting investigative agency who can commit to surveillance and collection of covert samples in accordance with applicable laws and policies [1];
    F. A representative from the prosecuting agency that can provide legal expertise [1]; and
    G. A program/project lead [1].
  4.2 Vendor laboratory vetting considerations
    Considerations for evaluating a vendor laboratory partner for the generation of the SNP DNA profile for FIGG may include type and status of accreditation, scientific and technical capabilities, and case circumstances. When feasible and considering case circumstances, FIGG should be conducted in a laboratory that operates under a quality assurance system and is ISO accredited [1].
    A. Technical review of data and interpretations by an independent qualified individual is a best practice included within accreditation.
  4.3 FIGG Memorandum of Understanding (MOU):
    Prior to conducting FIGG, an MOU may be established between law enforcement and prosecutorial agencies to acknowledge that any investigative leads that are provided will be followed up, charges laid, and actively prosecuted, if warranted [1]. The MOU may also include confidentiality, security, and data handling provisions. In some cases, genealogical accreditation of individuals may be based on experience and qualifications obtained prior to the development and acceptance of an official accreditation process.
  4.4 Genetic Genealogy Databases:
    A. FIGG should only be conducted using genetic genealogy database(s) (e.g., GEDmatch PRO and FamilyTreeDNA) that explicitly permits law enforcement participation and allows customers to choose their own privacy options (opt-in/out) of law enforcement comparisons for FIGG.
    B. Law enforcement agencies and forensic science service providers and their agents, including genealogical researchers and contractors, should abide by genealogy database providers' terms of service and privacy policies, and the consumers self-declared privacy settings.
    C. Law enforcement agencies and forensic science service providers recognize the role of genetic genealogy database providers as the responsible party to protect the privacy and confidentiality of all individuals within those databases, which includes individuals opting into law enforcement comparisons for FIGG, as well as those who choose to opt out. Law enforcement agencies and forensic science service providers and their agents, including genealogical researchers and contractors, will respect the privacy selections of all users, including those who choose to opt out of law enforcement comparisons for violent crimes using FIGG.
    D. Law enforcement agencies and forensic science service providers and their agents, including genealogical researchers, shall immediately notify the genetic genealogy database service provider of any error in code or process which may inadvertently expose individuals whose selected privacy options do not meet the terms of use for opted-in/opted-out kits when performing law enforcement comparisons for FIGG. The genetic genealogy database service providers shall immediately address such errors with corrective actions to address the root causes identified.
  4.5 Qualifications
    As noted above, the case under investigation is the responsibility of law enforcement and/or prosecuting agency. Private laboratories and private FIGG practitioners/providers are subcontractors to those agencies and the FIGG RA. Therefore:
    A. Qualifications of vendor laboratories will be determined by jurisdictional law and/or the FIGG RA with consultation of a DNA Technical Leader [9]. Determination of suitable qualifications may include accreditation status and compliance with jurisdictional requirements for processing forensic evidence.
    B. The laboratory conducting the SNP DNA profile generation and the genealogist participating in FIGG should be approved by the FIGG RA or prosecuting agency [9].
    C. Qualifications of FIGG practitioners should be determined by the FIGG RA [9]. Determination of suitable qualifications may include, but are not limited to, extensive FIGG experience, court deportment, and/or other qualifications that are reviewed and deemed suitable by the FIGG RA. FIGG Practitioners may be employed internally within an investigating agency or may be contracted externally with a private FIGG provider.
  4.6 Quality assurance
    A. Vendor laboratories should maintain documentation regarding their Quality Assurance Systems to be provided upon request [1].
    B. Vendor laboratories should compare SNP DNA profiles against a staff elimination database for contamination checks prior to the release of the SNP DNA profile to the agency [1].
    C. All laboratories conducting SNP sequencing for FIGG should be accredited. Acceptable standards include ISO-17025 or those deemed appropriate by the laws of the jurisdiction [1].
    D. FIGG practitioners should have successfully completed a competency test approved by the FIGG RA prior to conducting any FIGG casework.
    E. A quality assurance index search for contamination purposes may be conducted, including forensic and reference samples [1].
  4.7 Training
    FIGG education should be provided, by those with appropriate expertise, as follows:
    A. When a case is initiated to enable members of the FIGG program to better assist with the investigation, and
    B. With the release of FIGG investigative leads to investigators, as the lead may be a family member or not necessarily be the perpetrator,
    C. For prosecutors and justice system officials, and
    D. After the case is completed to all FIGG team members and the FIGG RA as lessons are learned [1].
    F. Defined and documented training should be provided to each FIGG team member commensurate with their roles and responsibilities [1].
5.0 Processes
  5.1 Data minimization and purpose limitation: Law enforcement should limit the collection, use, retention and disclosure of DNA and DNA-derived information to only what is reasonably necessary and relevant for a lawful IGG initiative.
    A. The FIGG RA should limit the collection, use, retention and disclosure of DNA and DNA-derived information to only what is reasonably necessary and relevant for the lawful provision of an investigative lead through FIGG. Information should be disclosed to individuals on a need to know basis only.
    B. There is no requirement for any health-related nor other types of sensitive information about known individuals who provide a genealogical database sample, beyond the relatedness of these individuals to the forensic sample. This includes physical characteristics, health issues, etc. As the identity of such an individual is already known, any further investigation of an individual who is determined not to be the perpetrator is to be considered an invasion of their privacy and could break the trust with them needed to provide LE access to their DNA kit to determine relatedness to help solve unsolved crimes.
    C. The identities, DNA, DNA-derived information, and other relevant information about individuals who are not the perpetrator should be protected and not used for any purpose beyond the investigation.
  5.2 Third-party DNA samples
    A third-party is an individual that is known not to be the individual who committed the crime, but rather one who may be genetically related and whose DNA sample may assist in building out further branches of a family tree or may directly lead to a candidate's identity through close relatives. General users (i.e., members of the public) populate the genetic genealogy databases, which are used to identify biological relative associations to forensic samples and can range from direct to close to distant relatives. These biological relatives provide a focal point from which to build out family trees. Through the building of family trees using genealogical records, a particular person may be reached in the tree(s) who it is believed may be genetically related to the forensic sample and may be a good candidate for third-party DNA testing.
    The collection of a third-party DNA sample from a discarded item from an individual is legal, however, where possible, it is strongly encouraged to obtain consent from the third-party. A State/provincial, regional, or agency's FIGG Programs policy should take into consideration the ramifications of covert sample collection from third-party/non-suspect individuals on public participation in and perception of FIGG. Maintaining the trust and goodwill of the general public who populate the genetic genealogy databases is of paramount importance to the successful use of FIGG, and the covert collection of samples from third-party/non-suspect individuals could undermine the trust which is depended upon by law enforcement for successful FIGG cases [15].
    Hence, the following non-exhaustive list of considerations may be evaluated with respect to the collection of third-party DNA samples to further genealogical research:
    A. Genealogical investigation has built family trees; however, it has reached an impasse where additional information is not forthcoming to continue reliably building the trees, and
    B. The collection of DNA samples from individuals identified through genealogical research could potentially breach the impasse by including or eliminating substantial portions of the family tree, and
    C. There are no other reasonable means to breach the impasse by conducting genealogical research without further DNA analysis, or
    D. The amount of genealogical research required to breach an impasse is so large, or so intrusive as to render third-party DNA analysis the most reasonable approach.
  5.3 Third-party DNA sample collection
    When the need for a third-party DNA sample has met a jurisdiction or agency's criteria, the collection of a third-party DNA sample may be considered when:
    A. Collection of a reference DNA sample from the third-party may provide additional leads to reach a candidate identification as noted above [8], and
    B. Written informed consent should be provided by third parties prior to obtaining a DNA specimen, unless:
      i. There is a concern that obtaining an informed consent sample from a third-party may jeopardize the integrity of the investigation, provide notification of a putative perpetrator that may endanger public safety, or cause the putative perpetrator to flee. If any of those scenarios exist, then covert sample collection may be pursued, if in compliance with jurisdictional, state/provincial, and federal laws and policies.
      ii. Covert collection is subject to the laws of the relevant jurisdiction for each investigation. Prosecutorial approval may be required or considered, depending on the circumstances.
    C. If a third-party denies providing an informed consent sample, consult with the prosecutor regarding potential next steps.
    D. Third parties should only be contacted by law enforcement conducting the collection rather than genealogists or forensic laboratory personnel.
    E. If overt collection of a third-party DNA sample is pursued, documented informed consent should be collected from the third-party [8].
    F. It is advised that a Non-Disclosure Agreement (NDA) should be considered so that the third-party does not discuss or distribute information regarding their participation in the case until provided with written notice that they may do so. An NDA may prevent interference in an ongoing investigation.
    G. It is also advised that the law enforcement officer/agent collects genealogical/family tree information from the third-party during interview to compare with the prior genealogical research conducted in the investigation.
    H. When a third-party has consented to participation, the third-party should be provided with options for how they can provide their DNA sample/data, and the relevant privacy options explained, which may include:
      i. If the third-party has previously taken a Direct-To-Consumer (DTC) DNA test (e.g., AncestryDNA, etc.), a request can be made to the third-party to voluntarily upload their DNA data file to the genetic genealogy database(s) themselves, or they may provide their downloaded DNA data file to the investigating agency for one-to-one comparisons to the forensic unknown DNA data [1]. Instructions should be provided to the third-party on how to download their DNA data file, and how to upload to the relevant genetic genealogy database(s). The relevant privacy options should also be explained regarding opting in/out for law enforcement comparisons. Third-parties should never be requested to provide access/log-in details to their DTC DNA test results at consumer DNA sites (e.g., AncestryDNA, etc.)
      ii. If the third-party has not previously taken a DTC DNA test, they may be offered to be supplied with a DTC DNA test (e.g., FamilyTreeDNA) by the investigating agency. The third-party should be informed of all the possible benefits and/or risks taking a consumer DNA test can have (e.g., matching to previously unknown close biological relatives, etc.). DTC DNA test results typically require 2–8 weeks to be processed. Once the results are ready, the third-party should be provided with further instructions to download/upload their DNA data file.
      If the third-party wishes to participate, however does not want to take a consumer DNA test, a buccal sample can be collected from the third-party for SNP sequencing analysis to generate a SNP profile for upload and comparison to the genetic genealogy database(s). The SNP profile should be uploaded as a “law enforcement upload” through the relevant portals/procedures to ensure their profile is not visible to any general users [1].
  5.4 Putative perpetrator DNA samples
    A. A putative perpetrator DNA sample that is collected covertly may be analyzed for a short tandem repeat (STR) DNA profile to determine if it matches an STR DNA profile obtained from the forensic sample.
    B. If judicial proceedings will be initiated, a direct reference sample should be obtained from the suspect by any legal means including, for example, upon arrest, with a search warrant, or with a court order. This direct reference sample should use STR analysis to confirm it matches an STR DNA profile obtained from the forensic sample.
    C. In the event that an STR DNA profile is not available for the forensic sample, a SNP profile may be generated for comparison between the reference sample and the forensic sample for the purposes of identification.
      i. In the event an STR DNA profile is not available, and a SNP profile is used for comparison to the forensic profile, the SNP data should not be used “to determine the sample donor's genetic predisposition for disease, any other medical conditions, psychological trait” [8,9], nor genealogical, medical, or scientific research of any kind.
  5.5 Uses of genetic information:
    A. Since the search and seizure of biological material abandoned at a crime scene is lawful, a forensic sample may be subjected to a wide variety of genetic analyses which can be developed to identify the donor, including phenotypic information and genetic ancestry.
    B. Forensic samples may be analyzed to provide potential eye color, hair color, skin color, and physical traits such as age estimation for the purpose of investigative intelligence.
    C. There shall be no use of reference samples beyond indirect and direct comparison utilizing STRs or SNPs. As by their definition, reference samples come from known individuals, hence there is little to be gained by use of these samples beyond comparison of some genetic markers to the forensic sample for the purposes of identification only. At the same time, the potential for other uses to undermine public trust is high.
    D. No data generated from the biological samples subjected to FIGG analysis, whether the forensic sample or third-party samples, may be used for other purposes such as “to determine the sample donor's genetic predisposition for disease, any other medical conditions, psychological trait” [8,9], nor genealogical, medical, or scientific research of any kind.
  5.6 Data management and security:
    A. The case under investigation is the responsibility of law enforcement and/or the prosecuting agency and not the vendor laboratory, nor the FIGG practitioner/provider who is performing research or analysis on the case. Therefore, responsibility for all documentation and data rests with the investigative authority and the FIGG RA.
    B. Data storage and transfer
      i. DNA data and case information should be stored in accordance with CJIS policies. A secure method for data storage, transfer, or exchange such as a secure file transfer protocol (SFTP) site should be established.
      ii. The FIGG RA should include policies that cover transitions, in the event a case is transferred from one laboratory or genealogical research entity to another.
      iii. Upon successful completion of the FIGG investigation as deemed by the FIGG RA (e.g., final judgment in a criminal case; or if no charges result, identification of a suspect or UHR confirmed by other means), “the [genealogist] participating in the [FIGG] shall turn over to the investigator all documentation, records, and materials collected, including material sourced from public records, family trees constructed, and any other genetic or nongenetic data analyses collected” [8,9].
      iv. If court testimony or additional analysis or research is required, appropriate documentation should be provided by the FIGG RA to permit analysis, investigation, preparation, and testimony as necessary.
    C. Data retention and deletion
      i. The retention and deletion of all FIGG data/documentation/records/trees built during a FIGG investigation must adhere to the corresponding country, state, provincial, and/or federal law.
      ii. DNA and DNA-derived information should not be retained longer than necessary. All DNA samples of all excluded persons of interest (POIs) must be destroyed without delay after the close of an investigation. Police should have retention protocols.
      iii. Each FIGG RA should have policy to safeguard all investigative information, data, family trees, and all other documentation generated through the course of the investigation. This policy should include information on what documentation should be generated; the form in which documentation should be kept; who has access; how information is to be shared, with whom and under what circumstances; and how long information is to be kept. All policy should conform to federal and provincial/state laws.
      iv. Case records should be kept as per a retention schedule and ideally to perpetuity as unsolved cases can be solved by linking to previously solved cases.
      v. SNP profiles developed from crime scene samples must be retained indefinitely, as a solved case may help solve an unsolved case where only SNP data is present and no STR to STR comparison is available [3]. Crime scene profile contributors are distinctly different from individuals who assist in solving the crime by providing access to their DNA to determine potential relatedness with the crime scene profile contributor.
      vi. Data from individuals known not to have committed the crime must be afforded full protection of data and identity. This includes those who have provided their samples to genealogical databases, individuals who have provided known samples to genealogical databases (known as third party or target tests) to assist in building family trees, and individuals who have provided elimination samples which have not matched the perpetrator.
      vii. It is recommended that all case data be maintained and retained by a single entity, backed up as per a regular and documented schedule, and held secure.
      viii. The FIGG practitioner/provider or vendor laboratory “may not keep any records or materials in any form, including digital or hard copy records” [9] unless legally required, as required by the agency's retention policy, and/or by a criminal justice agency [8,9] or FIGG RA.
      ix. If the DNA analysis was enabled by a third-party taking a direct-to-consumer (DTC) DNA test (e.g., AncestryDNA, etc.), and the third-party voluntarily provided their DNA data file for upload to the genetic genealogy database(s), then only the DNA analysis data possessed by the FIGG RA, investigating authority, forensic laboratory and genealogist will be destroyed.
      x. A person, agency or laboratory may not willfully retain or fail to destroy genetic genealogy information, SNP DNA profiles, DNA samples or DNA data generated during the course of the FIGG process that are required to be destroyed [9].
      xi. Once the FIGG has been concluded as determined by the FIGG RA, deletion or retention of third-party DNA samples and all associated data from their DNA analysis may be determined based on jurisdictional law or the agency's policy [8,9].
    D. Data disclosure and confidentiality
      i. No person should disclose genetic genealogy data, SNP DNA profiles, or DNA samples except where authorized by law or order of a court of competent jurisdiction or policies [8,9].
      ii. Identifying information of all third parties should be kept strictly confidential [8,9,16].
      iii. The prosecuting agency “shall retain and disclose any records or material as required under the [applicable state/provincial and federal regulation, the rules of discovery, or other court orders,] but may not otherwise use or share the records or materials” [8,9].
      iv. Neither the laboratory conducting SNP or other DNA analysis, nor a law enforcement official or a genealogist may disclose genetic genealogy information or details associated with an ongoing investigation without authorization from the prosecuting agency [8,9].
      iv. Consideration should be given by genealogical researchers to build family trees outside of public databases and their associated tools, as privacy, security, and confidentiality is not assured. For example, family trees in ancestry.com set to private are not assured to be so, therefore use of programs that reside on the private computer of a genealogical researcher are preferable.
      v. Personally identifiable third-party information may not be included in warrants and other legal documents which could reveal the identity of related individuals [1] noting these individuals are not suspects in the instant case and their privacy should be protected.
  5.7. Data-security: Law enforcement is expected to implement data security safeguard and measures.
    1. All police case files, documentation and investigative information shall be held secure with documented policies for security, access and release.
    2. Privacy must be maintained in all FIGG investigative case files to protect the identity of individuals who are not the crime perpetrator. This includes known individuals in genealogical databases who are related to the genealogical profile of the perpetrator or who have been included in family trees.
    3. All information contained in case records are to be held securely with access limited to those on a need-to-know basis.
  5.8. Specific controls for surreptitious DNA surveillance: Law enforcement should have safeguards on collection of DNA samples and must have reasonable grounds that a surreptitious DNA collection will afford evidence of a designated crime.
    1. The FIGG RA shall also have safeguards on the collection of known DNA samples, including reasonable grounds that the surreptitious collection of DNA will afford evidence of a designated crime.
    2. Safeguards for collection of known DNA samples shall be documented in a procedure which is followed for each collection.
    3. Adherence to documented procedure for the collection of known DNA samples shall be documented in the case file.
    4. The reasonable grounds used for collection of DNA shall be documented in the case file.
  5.9. Openness and transparency: Law enforcement should be open and transparent about their use of IGG including how they collect, use, retain and disclose DNA and DNA-derived information.
    1. The FIGG RA will provide information and education regarding the theory and use of FIGG.
    2. Included in the FIGG RA information and education is how DNA and DNA-derived information is collected, used, retained and disclosed.
    3. DNA and DNA-derived information shall only be used to provide investigative leads and in the investigation of criminal cases.
  5.10. Individual access and challenging compliance: Law enforcement should have a mechanism in place for individuals to challenge police compliance and the use of personal information including familial DNA and genealogical searches.
    1. The FIGG RA shall have a mechanism in place for individuals to file a complaint or challenge investigative compliance and personal information including familial DNA and genealogical searches.
  5.11. Public consultations: Law enforcement should conduct public consultation with affected communities, equity-seeking groups, and Indigenous communities.
    1. The FIGG RA shall conduct public education and provide information on the use of FIGG to affected communities, equity-seeking groups, and Indigenous communities.
    2. Public information and education materials shall be documented and available.
  5.12. Ethical disclosure guidelines: Law enforcement should develop ethical guidelines to inform their interactions with family members to avoid disclosing sensitive personal information.
    1. The FIGG RA shall develop and adhere to ethical guidelines to inform their interaction with family members to avoid disclosing sensitive personal information.
    2. Ethical guidelines shall be documented and available.
  5.13. Prioritization policy: All agencies should have a prioritization policy which is not driven by media or public opinion, but rather by facts surrounding the case and its standing relative to other cases. FIGG cases should be prioritized as follows and for the following reasons:
    1. Cases should be selected on merit if resources for FIGG are restricted.
    2. Cases should not be selected solely based on prosecution potential.
    3. Cases including unprosecutable cases should be considered for FIGG since cases that may be unprosecutable can be linked to other cases. Victims have a right to know and have their case solved, beyond what is prosecutable.
    4. Every UHR should be identified for the rights of the individual, family and loved ones.
    5. Conducting FIGG to solve case and identify individuals is in the public interest and includes the following considerations:
      1. Safety and security
      2. Humanitarian reasons
      3. Solving cases provides justice and resolution
      4. Better decisions are made with more information rather than leaving unresolved questions
      5. To become informed about prevention and better programs from past crimes and issues
  5.14. Testimony and Case File Retention: if vendors are utilized, procedures and MOUs should include ensuring the availability of staff to testify, even if they move to another company or leave the FIGG community. The case file shall be maintained and returned to the FIGG RA if the vendor discontinues business.
  5.15. SNP to SNP comparison: where an STR profile is not available for direct comparison, use of Identity SNPs should be used to confirm the identity as an investigative lead.
    1. It is recommended that a SNP to SNP crime scene profile database be started immediately to assist in solving unsolved cases where only a SNP profile from the perpetrator exists. This is only a recommendation at this time, as there will need to be law(s) passed in some countries including Canada to permit this. SNP to SNP comparisons could solve unsolved cases by linking them together [14]. If one case is solved by other means, then all cases with the same SNP profile will be provided this lead. Also, if one case is a partial SNP profile and another has a more extensive profile, and that second case is solved, then the lead generated by the second case could also be provided to the first case if the cases are linked through SNP profiles. While these instances may be low, because the data has been generated from the respective crime scenes, they are direct forensic to forensic comparisons. There may also be means to compare cases to each other to determine whether they are committed by related individuals. In summary, SNP-to-SNP comparison on unsolved crimes could create investigative leads where STR comparison is unavailable [14].
  5.16. Case file content: The FIGG case file including genealogical research must reflect sufficient information that another qualified individual can conduct a technical review and permit them to reach the same conclusion.
    1. Living individuals in a family tree must be included but can be redacted except for initials, such that a technical review can confirm who they are (see above).
    2. Individuals matched opt-in status and informed consent should be confirmed.
  5.17. Genealogical search tools: Genealogical search tools should be forensically validated to demonstrate the ability to identify matches, indicate their correct relationships to the FIGG sample, to document the analyses performed, and that they honor individuals' informed decisions to opt-in or opt-out.
  5.18. Documentation: Procedures for conducting FIGG should be documented.
    1. FIGG RA documentation should include the following procedures:
      1. Case and sample selection criterion
      2. Sample consumption policy
      3. Return of unconsumed sample and sample extracts
    2. The contents of a case file should be defined.
    3. The personnel who are provided access to what information and under what need-to-know circumstances should be identified.
      A. An audit should be conducted to ensure these individuals are following established policy and procedures.
      B. They should be accredited.
      C. Their training must include all of these points.
      D. Security of case files should be documented including how they are kept secure, and procedures for secure access.
    4. There should be restricted access to data storage facilities.
    5. There should be restricted access to data.
    6. Data should never be posted on publicly accessed websites.
    7. Family trees should not be built on publicly accessed websites.
    8. Results should not be shared unless approved by the FIGG RA.
    9. All training should be documented.
    10. Competency testing and ongoing proficiency testing should be documented.
    11. Policy and procedures should be documented.
    12. Case file documentation should be maintained in perpetuity.
    13. Verification of opt-in opt-out informed consent of individuals in the genealogical databases who are determined to be related to the forensic profile, and upon which the family tree is based that linked to the investigative lead.
  5.19. Discovery
    A. FIGG discovery must be provided in accordance with relevant law. The FIGG RA should disclose to a criminal defendant all material evidence collected during an investigation that has led to the filing of criminal charges. A defendant is entitled to examine information to determine its helpfulness without having to disclose how it might be helpful. Discovery can be met by providing copies of the documents themselves, or by providing access to examine documents.
      Historically forensic scientists have provided the information or access to information described in all of these categories during discovery to those responsible for prosecution who in turn provide access to defense, upon request if necessary. If FIGG has been conducted in a case, this information should be made available to defense whether or not the prosecutor intends to present the evidence. Investigators and prosecutors should receive all of the forensic science documentation or access to documentation before the case is presented for charging in order to review the materials and to decide what will be provided. The decision to provide these materials should not be left to the FIGG service provider. To avoid the hasty filing of charges with little time for review, discovery should be discussed with the team before the FIGG process begins.
      Providing the case file and/or access to materials in discovery affords the defendant due process to prepare their defense and to evaluate the strength of the case. The FIGG case file, including genealogical research must reflect sufficient information that another qualified individual can conduct a technical review and permit them to reach the same conclusion.
      Investigations using forensic science may include many sources of information that inspire confidence in the science applied to the case. These typically include:
      1. Documentation of the tests themselves,
      2. Qualifications of the scientists,
      3. Reliability of the techniques,
      4. Accreditation,
      5. Adherence to quality controls including proficiency testing,
      6. Compliance to industry oversight recommendations and standards,
      7. Other information including validation studies that inspires confidence in the proffered testimony without any showing that the material is necessarily helpful.
    B. As innocent individuals are documented in family trees through genealogical research that links a SNP DNA profile to biological relatives, who in turn are linked to the suspect, privacy interests of these individuals should be protected in compliance with jurisdictional law.
    C. If discovery is provided that includes identifying information associated with third-party individuals included in family trees, attempts may be made to protect this information such as with a court order.
    D. In the event there is no forensic STR DNA profile for a direct match to a suspect reference sample, a SNP panel will be required for comparison between a suspect reference sample and the forensic sample(s). It is anticipated that this situation will more likely require presentation in court and discovery should be provided in accordance with relevant law. For cases that do not require presentation in court, reports shall include sufficient information to be deemed informative by all interested parties.
    E. Adherence to policies and procedures can be enforced via procedures such as those provided by the FBI Quality Assurance Standards to ensure CODIS compliance [11].
    F. Items of information documenting the investigation including the forensic report(s) and case file for discovery must be kept available.
    G. The discovery items required may vary per jurisdiction, however some jurisdictions require everything be provided or available for inspection. Therefore, a court order should protect identities of everyone in the case file documentation. In particular, misattributed parentage may be seen in many case files and represents confidential information that may be incidental to the case. As redacted trees may not be an option, such sensitive information must be safeguarded and prevented from public disclosure, particularly if discovery is required.
    H. All data may be kept by the vendor but there should be an agreement that the data will be made available and turned over the FIGG RA, and that a data retention plan be included to protect case data and files in case of vendor bankruptcy, buy out, or other circumstances that prevent the vendor from continuing FIGG operation.
    I. There should be confirmation of genealogical matches have opted-in to the genealogical database.
  5.20. Reporting
    Data that can aid in the continuous improvement of the FIGG program should be collected [8,9]. Data may include the following:
    A. Number of FIGG cases investigated [9];
    B. Type of crime for which the FIGG case is being investigated;
    C. DNA specimen details including type of specimen (biological material), DNA quantity and quality;
    D. Type of DNA typing and analysis applied to the sample specimen;
    E. Identity of vendor laboratories conducting SNP sequencing analysis and FIGG practitioners/providers conducting genealogical research;
    F. Number of FIGG cases entered into genetic genealogy databases;
    G. Number of leads resulting in an identification [8,9];
    H. Costs of FIGG procedures (both cumulative and categorized by laboratory workflow (in-laboratory or vendor), genealogy, investigation [8,9];
    I. “Number of times a third-party DNA sample was requested and collected” [9];
    J. “Number of [FIGG] requests made by defendants and post-conviction attorneys” [9]; and/or
    K. The case outcomes of each FIGG investigation [8,9].
  5.21. Oversight
    The FIGG RA, forensic laboratories, and investigative agencies implementing FIGG may choose to consult with a panel, a commission, or a committee. The panel, commission, or committee shall be comprised of stakeholders deemed appropriate by the relevant jurisdiction.“A panel comprised of judges, prosecutors, defense attorneys, public defenders, law enforcement officials, crime laboratory directors, bioethicists” [9], genealogists, “racial injustice experts, criminal justice researchers, civil and privacy rights organizations, and organizations representing the families impacted by the criminal justice system” [9], including victims’ rights advocates, “may be convened to review the annual report each year and make policy recommendations” [9].
  5.22. Data Retention Recommendations for Sequencing Data
    1. At a minimum the following data should be retained for FIGG cases:
      1.1 Post-demultiplex files, prior to bioinformatics/imputation/mixture deconvolution should be retained such that the data can be reanalyzed. (These files may be FastQ’s or BCL files for example.)
      1.2 Final outputs for upload, ie GEDmatch Pro and FTDNA upload files (text).
      1.3 Bioinformatic pipeline version information should be captured to facilitate re-analysis using the same conditions as previously utilized.
    2. At a minimum the following data should be retained for non-FIGG forensic cases:
      2.1 Files prior to bioinformatics/imputation/mixture deconvolution should be retained such that the data can be reanalyzed. (These files may be FastQ’s or BCL files for example.)
      2.2 Record of edits made.
    3. Storage TimeData shall be retained, at a minimum, until the case is considered closed, and any possible appeals processes have been exhausted. Jurisdictional guidelines should also be adhered to regarding duration of storage. At the request of the client or by court order the data may be purged. Additionally, if the data is transferred to the client, the laboratory is absolved from retaining the data as long as all parties are in agreement, and it complies with local law.
    4. Third Party AccessAccess will only be granted as result of a court order or under the express permission of the requisite parties, i.e. for a criminal profile from the law enforcement agency and for a reference profile from the donor.

## 4 Modifications from previous version

1. In Section 2, new sections 2.1 Lawful authority and activity and 2.2 accountability added.
2. In Section 3.0, new sections 3.1 through 3.5 added to address necessity and proportionality. Expand to include country and province. Renumber remainder of section 3, now 3.6 through 3.9.
3. In Section 3, new section 3.10 Third Party procurement added.
4. In Section 4, 4.2.A. Technical review is added.
5. In Section 4.3, additional wording noting individual accreditation may be based on previous experience and qualifications.
6. In Sections 4.4, 4.5, 4.6, and 5.1 instances of “shall” replaced by “should” as this a document of recommendations rather than a standard.
7. In Section 5, new sections 5.1 Data minimization and purpose limitation and 5.7 Data Security added. Renumber remainder of section 5.
8. In Section 5.6.C Data retention and deletion, update 5.6.C.i adding country and provincial.
9. In Section 5.6.C.ii, add new points ii through vii. Renumber remainder of 5.6.C.viii. through xii.
10. In Section 5.6.c.xi, remove this section regarding deleting family trees build in publicly available databases, as it is redundant due to Section 5.6 recommending family trees not be built in publicly available databases or shared software.
11. In Section 5, add new sections 5.8 Particular controls for surreptitious DNA surveillance, 5.9 Openness and transparency, 5.10 Individual access and challenging compliance, 5.11 Public consultations, 5.12 Ethical disclosure guidelines, 5.13 Prioritization policy, 5.14 Testimony and case file retention, 5.15 SNP to SNP comparison, 5.16 Case file content, 5.17 Genealogical search tools, 5.18 Documentation and 5.22 Data retention recommendations for sequencing data. Renumber remainder of 5.19 through 5.22.
12. In Section 5.19 Discovery, additional material added under 5.19.A, and addition of points F through I.
13. Update references section adding references.

## CRediT authorship contribution statement

**Ray A. Wickenheiser:** Project administration, Supervision, Writing – original draft, Writing – review & editing. **Jennifer Naugle:** Writing – review & editing, Writing – original draft. **Brian Hoey:** Writing – original draft, Writing – review & editing. **Rylene Nowlin:** Writing – original draft, Writing – review & editing. **Claire Glynn:** Writing – original draft, Writing – review & editing. **Raymond Valerio:** Writing – review & editing, Writing – original draft. **Lance Allen:** Writing – review & editing, Writing – original draft. **Stephanie Stoiloff:** Writing – review & editing, Writing – original draft. **Jennifer Kochanski:** Writing – original draft, Writing – review & editing. **Mary Beth Schubert:** Writing – original draft, Writing – review & editing. **Melissa Haas:** Writing – review & editing. **Dixie Peters:** Writing – review & editing. **Rachel Oefelein:** Writing – review & editing. **Rockne Harmon:** Writing – original draft, Writing – review & editing. **Barbara Llewellyn:** Writing – review & editing. **Trisha Kacer:** Writing – review & editing. **Blake Patel:** Writing – review & editing. **Paul Panther:** Writing – review & editing. **Colleen Fitzpatrick:** Writing – review & editing. **Garry Bombard:** Writing – review & editing. **Alison Sears:** Writing – review & editing. **David Mittelman:** Writing – review & editing. **Stacey Baker:** Writing – review & editing. **Stephanie M. Fullerton:** Writing – review & editing. **Jessica Koong:** Writing – review & editing. **Meredith Chambers:** Writing – review & editing. **Melisa Wittkowske:** Writing – review & editing. **Ken Carrierre:** Writing – review & editing. **Nichole Schwedelsky:** Writing – review & editing. **Ted Liomanis:** Writing – review & editing. **Michael Kelly:** Writing – review & editing. **Stephen Smith:** Writing – review & editing. **Jonathan Nauman:** Writing – review & editing. **Tom Cober:** Writing – review & editing.

## Declaration of competing interest

The authors declare the following financial interests/personal relationships which may be considered as potential competing interests:

Dr. Ray Wickenheiser conducts forensic consulting and has provided services to Othram and Qiagen independent of this paper.

Melissa Haas is currently employed by Bode Technology.

Dr. David Mittelman is an employee of Othram Inc and there are no other competing interests or relevant affiliations with any organization or entity with the subject matter or materials discussed in the manuscript apart from those disclosed.

Dr. Rachel Oefelein is employed by DNA Labs International, a private forensic testing laboratory providing services to law enforcement, attorneys, and government laboratories.

Jessica Koong is an employee of Qiagen (GEDmatch).
